# Human Schistosomiasis Resistance to Praziquantel in China: Should We Be Worried?

**DOI:** 10.4269/ajtmh.2011.10-0542

**Published:** 2011-07-01

**Authors:** Edmund Y. W. Seto, Betty K. Wong, Ding Lu, Bo Zhong

**Affiliations:** School of Public Health, University of California, Berkeley, CA; Institute of Parasitic Diseases, Sichuan Center for Disease Control and Prevention, Chengdu, Sichuan, China

## Abstract

The efficacy of praziquantel for the treatment of *Schistosoma japonicum* in humans is reported from a cross-sectional survey conducted in 33 villages in Sichuan Province. Infection prevalence was found to be 5.7% (185 infected of 3,269 tested) in a region where 44–73% prevalence was found 9 years before. Collected miracidia were subjected to an *in vitro* test of praziquantel susceptibility. An effective concentration of praziquantel associated with 50% of miracidia changing shape was found between 10^−8^ and 10^−7^ M and 10^−7^ and 10^−6^ M for 10 and 5 minutes of exposure, respectively. After treating infected persons two times with 40-mg/kg doses of praziquantel, only one remained infected. Findings are reported from a 60-household questionnaire on attitudes and behaviors that may be associated with development of drug resistance. The low number of treatment failures and good compliance with treatment despite side effects and repeated annual treatments suggest that, in the near term, praziquantel remains effective in treating human *S. japonicum* infection in China.

## Introduction

Praziquantel is used to treat all forms of schistosomiasis. The drug was introduced in clinical practice in the late 1970s, and its chemistry, clinical efficacy, and side effects have been extensively reviewed.^1^,^2^ The antischistosomal effects of praziquantel in animals were published in 1977, and the effects in humans were published in 1978.^3^–^5^ Early clinical trials carried out for *Schistosoma japonicum*, *S. mansoni*, and *S. haematobium* found praziquantel to be highly effective across a wide spectrum of schistosomes.^6^–^8^ Additionally, on a per-case basis, treatment with praziquantel is inexpensive. It currently costs around US$0.40 or less to treat a case of schistosomiasis.^2^ All of these factors have made praziquantel treatments one of the most important strategies to reduce the morbidity associated with chronic schistosomiasis.

Before the widespread use of praziquantel (called pyquiton) in China, the national control program for schistosomiasis established in 1955 focused largely on interrupting transmission of infection through environmental management and identifying strategies that would work in different regional settings (the plains, mountainous, and hilly areas versus marshland and lake regions).^9^ Control strategies consisted of snail control, identifying infected humans and other animal reservoirs, and use of antimonials such as potassium antimony tartrate or fouadin initially and later, the non-antimonial furapromidum. The antimonials were quite toxic, and the non-antimonials had suboptimal safety profiles. Therefore, none of these drugs were used for large-scale chemotherapy.^10^

Praziquantel saw widespread use in China during the 10-year World Bank Loan Program starting in 1992.^11^–^13^ Large-scale administrations of praziquantel were implemented for both human and bovines for morbidity control with the aim of reducing *S. japonicum* prevalence in both populations by at least 40%. Different strategies were used in different areas for chemotherapy administration depending on regional differences in infection prevalence: mass treatment of the entire population aged 5–65 years without preliminary screening for high-endemic regions, selective treatment of persons identified through diagnosis to be infected in medium-endemic regions, and phased treatment using mass and selective treatment in sequence as regions transition from high to lower endemicity.^10^ In many cases, chemotherapy was combined with other key strategies to sustain transmission control, including health education and snail control through environmental management and limited mollusciciding. The program resulted in dramatic reductions in schistosomiasis in China.^11^–^13^

Current recommended human dosages of praziquantel to treat schistosomiasis are based on weight of the human host (40 mg per kg body weight). Using recommended dosages, cure rates of 80–90% have been documented for *S. japonicum*.^14^ However, schistosomes are generally only sensitive to praziquantel during their earliest stage as schistosomula, which is a few days after infection, and then, during their later stage beginning around weeks 6–7 after infection.^1^ The lack of efficacy against juvenile schistosomes has lead to development and occasional but rare use of other drugs, such as artemether, which is effective against the juvenile stages of the parasite.^11^,^15^

Little evidence exists on the potential praziquantel resistance of *S. japonicum*. Ross and others^11^ state that, despite minor side effects (nausea, dizziness, rash, and pruritus), there have been no fatalities associated with praziquantel treatment, and there has not been unequivocal evidence that schistosomes have developed drug resistance. Cure rates are relatively high (70–90%), with those not cured still benefiting from reduced egg counts.

There is evidence to suggest that *S. japonicum* may be generally more susceptible to praziquantel than other schistosome species.^16^ However, factors such as widespread and indiscriminant use of praziquantel with little prior diagnosis and the potential for decreasing compliance with drug dosages because of treatment fatigue, poor education, and indifference to chronic infection suggest the need for careful consideration as to whether praziquantel resistance may be developing in China.

Recent studies have established an *in vitro* test of potential praziquantel resistance based on computing the effective concentration at which 50% of the miracidial form of the parasite becomes inactivated (EC_50_).^17^ The method has shown higher EC_50_ levels for purported difficult-to-treat strains of *S. mansoni*. Despite laboratory assessments of resistance to praziquantel, there remains debate whether resistance results in any practical challenges for everyday disease control efforts.

In this paper, we present the results of a cross-sectional infection survey conducted in 33 villages in an endemic region of Sichuan, China, which has undergone intense chemotherapy treatment for nearly a decade. Miracidia hatched from infected individuals' stool samples were subjected to an *in vitro* assay of praziquantel susceptibility. Additionally, infected persons were treated and retested for infection to determine the proportion of treatment failures, which is another indicator of resistance. Based on a survey of 60 households, we document some of the factors that may contribute to resistance and treatment failures in this region of China.

## Methods

### Study area.

Our study was conducted in one township within a schistosomiasis-endemic county of southwest Sichuan province, which is representative of the hilly and mountainous transmission ecology of China. The township consists of three administrative villages that are further divided into 33 natural villages, which collectively account for a population of approximately 5,860 persons (based on official resident records). Administrative villages are subdivided into natural villages, also known as production groups, which are the smallest level of organization in rural China. The majority of the population is farmers, who grow tobacco and flowers in the spring season and wheat and beans in the fall season, although aquaculture is prevalent in some villages.

Previous research conducted in this region has documented the risk factors associated with transmission, including the relationships between infection and cercarial exposure and other environmental determinants of transmission.^18^,^19^ Four of the current selections of natural villages were part of the previous study. Increasing use of chemotherapy to reach disease control targets has motivated our group to revisit this study area to assess praziquantel compliance and efficacy. In 2000, the prevalence of infection as determined by positive result on either the miracidial hatch or the Kato–Katz examination in 4 of 33 natural villages in the township was found to range from 44% to 73%.^18^ After treatment, in 2002, a reassessment of infection found that prevalence had decreased to 23–38%.^19^ Successive chemotherapy administrations, snail control, and introduction of biogas into the region have resulted in the declaration of transmission control in the region (all of Sichuan Province) in 2008. The national criterion for transmission control is defined as < 1% infection in human and cattle, no new infections in children and young cattle, no acute cases, and snail habitat reduced by 98% of historical levels. Our cross-sectional survey described below provides an independent confirmation of the control achievement. At the request of our Chinese collaborators, who are sensitive to the release of detailed infection data from a region that has been officially declared to have attained transmission control, we have withheld the names and exact locations of the villages in this study.

### Parasitological examinations.

In November of 2009, all individuals aged 6 years and older from the 33 natural villages (also called production groups) in the study area were asked to participate in a cross-sectional infection survey. With written informed consent, a stool sample was collected from each participant. The stool was first examined for presence of infection using the miracidial hatch test. Briefly, the hatch test consists of straining 30 g of a subject's stool through nylon mesh to isolate parasite eggs, which are then incubated in an Erlenmeyer flask of dechlorinated water at 28–30°C and observed at regular intervals for up to 8 hours for the presence of hatched miracidia. For individuals found to be hatch test-positive, a subsequent Kato–Katz fecal smear test (for each person, one homogenized stool sample and three 41.7-mg slides) was conducted to quantify egg excretion (in units of eggs per gram of feces [EPG]).^20^

All infected individuals were treated with two times the recommended dosage of praziquantel (40 mg/kg) for approximately 2 and 3 weeks after the infection survey. After an additional week (approximately 1 month after the initial infection survey), individuals were retested for infection using the hatch test. Those found to be infected were retreated until infection was not found. Although praziquantel has a biphasic efficacy period and it has been recommended that treatments be staged 3 weeks apart, in our case, the examinations were conducted in the winter season when snails do not shed cercariae, transmission is negligible, and hence, infection with juvenile parasites unlikely.^21^ Furthermore, because transmission is negligible, residual infection is likely to result from treatment failure rather than advancement of new juvenile infections.

### *In vitro* praziquantel assay.

Based on the methods of Liang and others^16^ and Botros and others^17^, a miracidial assay was used to determine the effective concentrations of praziquantel at which 50% of the parasites would change shape (EC_50_), an indicator of sensitivity to the drug. Because of low numbers of parasites per host sample, all available miracidia were collected from the hatch tests of all infected individuals and combined. Miracidia were placed into 24-well flat-bottom microplates.

A praziquantel solution was prepared by dissolving praziquantel (Nanjing Pharmaceutical Factory, Nanjing, Jiangsu, China) in dimethyl sulfoxide (DMSO) to create a 10 mM stock solution. Serial dilutions were performed to create solutions at 10^−8^, 10^−7^, 10^−6^, and 10^−5^ M concentrations. Because the number of miracidia was generally low, we were only able to test small numbers of miracidia at each concentration. At least five miracidia were exposed for 10 minutes at each concentration. At least 20 miracidia were exposed at the two middle concentrations. Miracidia were also exposed with two controls: praziquantel-free 10^−3^ M DMSO and decholorinated water. Miracidia were observed at 1-minute intervals, and the number that underwent morphological changes indicative of sensitivity to praziquantel was counted.^16^

### Praziquantel resistance risk factors survey.

Twenty household surveys were conducted in each of the three administrative villages (60 total surveys; natural villages 1 and 6 and one each in administrative villages 1, 2, and 3, respectively) to ascertain social and cultural factors that relate to aspects of villagers' perception: the role of key players in schistosomiasis diagnosis and treatment, the experience with praziquantel drug therapy; knowledge of schistosomiasis in terms of infection and prevention, access to and use of clean water and latrines, and contact with unsanitary water. Households were recruited randomly based on the availability of household members during the time surveys, which was a weekday morning in January. One person was selected from the household to complete the survey.

The first section of the survey assessed the roles of the schistosomiasis control station, village leader, villager doctor, family, and school when villagers become infected and need diagnosis or treatment. The second section assessed villagers' experiences with praziquantel by understanding their drug treatment compliance, others who may play a role in their compliance, the convenience of acquiring the drug, their experience with side effects of the drug, and whether the side effects affect their daily lives. The third section evaluated villagers' knowledge of schistosomiasis transmission and prevention methods and also asked which prevention methods, if any, that they used. The fourth section investigated water and sanitation with two subsections: presence of latrines and biogas systems and access to clean water versus contact with unsanitary water.

Ethical approval for this study was provided by the University of California Committee for the Protection of Human Subjects, and written informed consent was obtained for participation.

### Statistical analyses.

Between-group differences in infection prevalence and between-group survey responses were assessed by Fisher's exact test. To assess the associations between infection and various factors related to schistosomiasis perception, drug treatment experience, knowledge of transmission, and exposure, logistic regression was used to estimate the odds ratio (OR) of self-reported history of infection as a function of the responses to the questions in the risk factors survey. History of infection was defined as an affirmative response to the question “Have you previously been infected?” We used history of infection rather than current infection because of the small numbers of current infections in the 60 household samples. Models were fit individually to each survey question variable, unadjusted and adjusted for age, gender, and income. Within administrative village, correlation was accounted for using generalized estimating equations (GEE) with exchangeable correlation and inference from robust variance estimates. *P* values less than 0.05 were considered statistically significant. Analyses were conducted using Stata v.10 (StataCorp, College Station, TX).

## Results

### Infection results.

**Figure 1.**
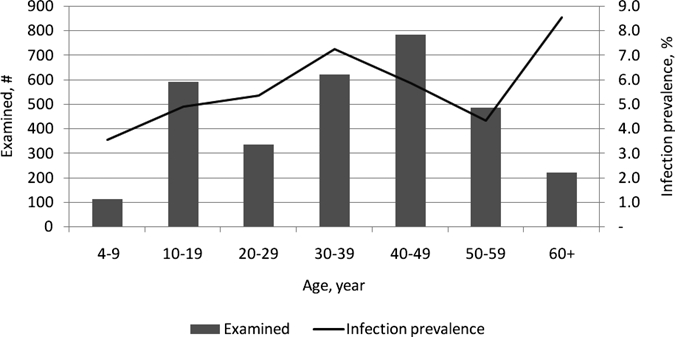
Age distribution of participants and infection prevalence.

Of the registered village residents, 5,862 were eligible for the study, and 3,269 (56%) participated in the infection survey. The low participation rate was expected because of individuals leaving their village to work in cities. Generally, the age of the missing individuals was centered at 25 years of age. Of the study participants, 185 (5.7%) were found to be infected. Of those that participated, 3,153 had complete age, sex, and infection data, which are summarized in Table 1 and Figure 1. Although there were relatively low numbers examined in the lowest age group (< 10 year olds), they had the lowest levels of infection. Conversely, the highest infections were found in the 30–39 and 60+ age groups. Infection tended to be higher in males than females for most age groups, except most notably for the 40–49 year olds.

Infection prevalence for natural villages ranged from 0% to 17% (Table 2), with prevalences of 7.7%, 2.6%, and 5.9% in administrative villages 1, 2, and 3, respectively. Prevalence of infection was notably lower in the four natural villages for which historical data were available from 2000 and 2002 (*P* < 0.001).

The Kato–Katz examination had less sensitivity to detect eggs than the hatch test. Of the 185 found to be hatch test-positive, 177 participated in the Kato–Katz examination, and 108 (61%) were Kato–Katz-negative. Overall, mean egg excretion was 22 EPG (standard deviation [SD] = 97, range = 0–1,263), whereas for those with positive Kato–Katz results, the mean excretion was 58 EPG (SD = 151, range = 8–1,263).

After treatment with two 40-mg/kg doses praziquantel, only 1 of 185 original hatch test-positive persons was found to still be hatch-positive on reexamination after 1 month. After a second treatment with two 40-mg/kg doses praziquantel, the person was no longer found to be infected.

### Miracidial assay results.

Table 3 shows the results of *in vitro* tests of miracidial susceptibility to different praziquantel concentrations and exposure times. Susceptibility was determined by a characteristic shape change that occurs to the miracidia when exposed to praziquantel as described by Liang and others.^16^ A relatively small number of parasites was available for the *in vitro* test because of the low levels of infection and miracidia recovered from hatch tests. At each concentration, the percent of normal (shape unchanged) miracidia generally decreased with exposure time, except in the control groups. Also, for each exposure time (column), the percent of normal miracidia decreased with higher concentrations. The EC_50_ was found to lie between 10^−8^ and 10^−7^ M for 10 minutes of exposure and 10^−7^ and 10^−6^ M for 5 minutes of exposure, respectively. At the highest concentration, one miracidia was found to remain unchanged up to the maximum of 10 minutes exposure.

### Resistance factors survey results.

Sixty household surveys were completed. The demographics of the participants are presented in Table 4 for each of the three administrative villages. Generally, the age, economic status, educational attainment, and occupations of participants were similar across villages (*P* > 0.05), although a larger proportion of females participated in one of the villages (*P* = 0.025 between villages 1 and 3) and another village included participation of those with business occupations (typically, those operating small stores).

The assessed risk factors related to potential development of praziquantel resistance are summarized in Table 5. Although there were statistically significant differences in the responses related to roles and responsibilities for schistosomiasis diagnosis and treatment across administrative villages, generally, most stated that the schistosomiasis control station had the greatest responsibility, despite the availability of praziquantel at rural village doctor's clinics. Despite greater responsibility assigned to the control station, roughly two-thirds of respondents reported that they seek treatment from the village doctor, and roughly one-third reported that treatment is obtained from township leaders, who are often recruited by the control station to administer drug treatments to their residents. Moreover, the majority responded that the local village leaders played an active role in reminding villagers to take the drug.

Roughly one-half of respondents reported that they had been previously infected with schistosomiasis, which is expected given that the township historically has been a high-endemic area. The differences in self-reported infection history between administrative villages were significant (*P* = 0.015). However, self-reported infection was higher and rank was ordered differently for villages than current infection levels assessed from the hatch tests. This suggests that drug treatment has been quite intense in this area, but reinfection has occurred differentially between villages. There was no significant differences between villages in the amounts of treatment in recent years, with the majority of participants reported taking the drug three times in the past 3 years, which corresponded to the schistosomiasis control station's annual chemotherapy administrations aimed at reaching transmission control status in the province.

Participants reported that the drug is readily available and reported good drug compliance, with nearly all reporting that they take the entire dose of drug. Most took the drug immediately, and those that did not reported that they took the drug at night before bedtime, which is sometimes recommended by control staff to alleviate side effects. Roughly one-half of respondents experienced some side effects from praziquantel. The reported symptoms were generally mild (lightheadedness, vomiting, stomach ache, dehydration, and chills). However, one-third of respondents reported that these effects were serious enough to affect work. The reporting of side effects that affected work was strongly positively associated with number of previous treatments in the past 3 years (OR = 335, *P* < 0.001, 95% confidence interval [CI] = 299–376). In addition, we found that males were less likely to report side effects (OR = 0.32, *P* = 0.003, 95% CI = 0.15–0.68). There were no significant differences in reported drug treatment behavior or side effects between villages.

The responses to the questions about knowledge and prevention of schistosomiasis as well as questions about clean water and sanitation suggest that schistosomiasis reinfection may continue to be a problem in this area. Despite most reporting that contact with unsafe water is associated with infection, more than one-third of participants incorrectly reported that transmission had to do with drinking unclean water, and another 8% reported that they did not know how infection occurred. Additionally, approximately one-quarter of respondents felt that there was nothing that they could do to avoid infection. Despite all participants reporting that they had access to clean piped water, contact with unsanitary water sources was still high for a variety of common activities. Also, the majority of participants felt it was acceptable to defecate outdoors in the wild.

There were promising responses in terms of the potential for rural development and environmental modification to reduce the risk of infection. Nearly one-half of respondents reported having biogas toilets, which has been previously found to reduce the risk of schistosomiasis as well as be highly accepted by villagers.^22^ Additionally, there was very good understanding of the potential role that bovine populations play in schistosomiasis transmission, which led to many suggesting that removal of cow stool from the environment may be a worthwhile endeavor.

To gauge the impact of schistosomiasis treatment, perception, knowledge, and behaviors on infection, we estimated the unadjusted and age-, gender-, and income-adjusted OR of self-reported historical infection for each survey question (Table 6). Although treatment side effects and drug-taking behavior were not significantly associated with historical infection, the OR of historical infection was lower among those that relied on local village doctors than the control station. Conversely, the OR was higher for those that had a better knowledge of one of the preventative measures (gloves; e.g., when washing clothes or vegetables in potentially infective ditch water), with the positive correlation perhaps suggestive that prevention health education messages have been effectively delivered to individuals at greatest risk. Finally, the highest ORs were estimated for various exposure factors, including contact with ponds and contacts related to cooking and drinking, washing vegetables, and bathing activities, suggesting that continued exposure remains a large driver of ongoing infection.

## Discussion

This study presents data on current *S. japonicum* susceptibility to praziquantel and risk factors for the development of drug resistance in a region of China where transmission control was recently attained through intense chemotherapy. We found considerably lower prevalence and mean intensity of infection than in previous surveys conducted in 2000 and 2002. Based on infectious disease modeling work, we feel that the dramatic reductions in infection largely have been because of annual chemotherapy but also because of control of snail populations and complementary policies and interagency coordination, which have allowed the introduction of improved biogas sanitation to this area.^23^ Although the county as a whole has achieved the status of transmission control, in our 33 study villages, the infection prevalence was 5.7%. This is, however, still quite low. Of the 185 persons found to be infected and treated, only one failed treatment was suspected. This is remarkable considering the cure rates of 80–90% reported in the *S. japonicum* literature.^14^ The high efficacy may be in part because of the low levels of infection in this region or infection being reduced to undetectable low levels.

The epidemiologic patterns of infection in our study reflect the current transmission situation and ongoing control challenges found in Sichuan. Although infection was lower than in previous studies in this area, the patterns of infection with age were similar, with the prevalence of infection not highest in the youngest age groups but rather, in mid-aged adults and older populations. Participation for the young adult age group was low, because many of these people no longer live in their villages but spend large amounts of time away working in cities. This is a phenomenon that we have seen in recent infection surveys conducted in other parts of the province, and we are actively studying them to determine the extent to which this population group may be harboring infection, and if so, whether they may reinitiate infection in controlled areas when they return to their villages. Additionally, some of the infection statistics in our study reflect the diagnostics that we used. For example, in our study, the Kato–Katz failed to measure eggs in 61% of people who had tested positive in the hatch test. This strongly suggests the need for better diagnostic tools if we are to continue to track the efficacy of chemotherapy and other types of control activities in low-transmission settings.

Our *in vitro* tests of miracidia found limited evidence for reduced parasite susceptibility to praziquantel. A small number of parasites were found to survive prolonged and high concentrations of praziquantel. Additionally, comparing our EC_50_ results with those of Liang and others^16^ suggests that Sichuan parasites may have less susceptibility to praziquantel than those found in other provinces.^16^ For example, for Hunan province parasites, at 5 minutes of exposure, 0% and 64% of miracidia were unchanged for concentrations 10^−6^ and 10^−7^ M. For Jiangxi province parasites, 0% and 60% of miracidia were unchanged for the same exposure and concentrations. For Hubei province parasites, 0% and 63% were unchanged. We found 29% and 71%, although we tested much smaller numbers of miracidia. The fact that only one treatment failure was suspected from our epidemiologic study suggests that laboratory evidence of diminished sensitivity to praziquantel may have negligible effect on real-world efficacy of control programs in this region of China but again, with the caveat that this population had very low-level infection.

Our *in vitro* study was limited by the numbers of miracidia that we were able to recover from the hatch tests. Previous studies have relied on either higher infection areas and/or laboratory-maintained lifecycles to obtain larger numbers of miracidia for such work. Despite our low sample numbers, we found relatively consistent concentration response profiles for increased susceptibility with increasing exposure time and increasing concentration of praziquantel.

Our laboratory work reflects the need for a relatively simple assay of parasite changes that may be occurring from praziquantel treatments. Although we lack evidence of this, treatment may also be responsible for functional changes in the parasite population (e.g., impaired mating, reduced fecundity, greater susceptibility to within-host density dependence, or less competent larval stages, all of which may be responsible for temporal changes in epidemiology that we have seen in our field site). Given the continued reliance on chemotherapy, a greater understanding of the occurrence of these population changes is warranted. Related work by Lamberton and others^24^, who used the same *in vitro* assay on *S. mansoni* isolates, provides further motivation for the routing use of the method as a screening tool to identify population structure changes in schistosome-endemic regions where control programs are operating.

Although our risk factors survey recruited households randomly from each of the three administrative villages in the township, participation was likely biased based on the availability of the villagers. Because of limited field resources, the number of participants in our survey was low. However, we had participation from both genders as well as a wide range of ages and a good representation of farmers, which constitute the predominant occupation and risk group in the area. Because of this, we feel that the responses generally reflect the knowledge, attitude, behavior, and experience of villagers in this region, including those with a history of infection, because more than one-third of respondents reported previous infection.

The responses from the risk factors survey may have several implications for the development of praziquantel resistance over time. First, chemotherapy treatments have been intense, with almost all respondents reporting annual treatments, which may create treatment fatigue and non-compliance over time. Although availability of the drug and compliance still seem high, there is concern that the high rates of side effects may further contribute to non-compliance in the future. Although other studies have found evidence of harsher side effects of praziquantel treatment with high intensity infections, our finding of roughly one-half of respondents reporting mild side effects is consistent with other *S. japonicum* studies conducted on low- to middle-intensity infection populations.^1^,^11^,^25^ For example, adverse effects were found in 58% of light infections in Japanese patients (treated with three 20-mg/kg doses).^6^ Santos and others^26^ found similar percentages of adverse reactions (53.3% and 61.5% for 3 × 20-mg/kg treatment of light infections and patients with hepatosplenic involvement, respectively) in the Philippines.^26^ In China, Wu and others^27^ found adverse side effects in 62% of treated patients, which is higher than we found. However, their study population had higher pre-treatment infection intensity (as measured by EPGs) than our study. The previous Japanese, Philippines, and Chinese studies also found that side effects tended to be mild. Despite generally mild effects, of particular concern for ongoing treatment compliance is our finding of dramatically higher rates of more adverse work-affecting effects reported among those that had received more treatments in the last 3 years and the higher rate of effects among some risk groups (females).

The roles and responsibilities for diagnosis and treatment vary between parties. We found that much of the responsibility was placed on the schistosomiasis control station, but some key tasks were placed on village doctors (treatment and reducing infection) and local leaders (treatment and reminding villagers to take the drug). Our finding of an inverse relationship between self-reported history of infection and perceptions of village doctors' role in treatment and prevention confirms the importance of control station activities in reducing infection in highly endemic populations, but it also suggests an opportunity for local entities to play a larger role in control. These entities must work in concert to make sure that all infected individuals comply with infection testing and drug administration if treatment failures are to be minimized.

The survey responses indicate gaps in schistosomiasis health education and poor attitudes to making behavioral changes to reduce exposure and contamination, which may contribute to ongoing infection. However, some preventative health education messages, such as wearing gloves (e.g., to avoid parasite exposure during clothes- and vegetable-washing activities) seem to have been understood by the previously infected populations. Despite access to clean water among all participants in the survey, contact with unsanitary water (irrigation ditches, lakes, and ponds) remains high and is perhaps inevitable given the rural agrarian lifestyle in this region. Notably, current exposure to unsanitary water through a variety of activities remains high among those that have been historically infected. Rural development in the form of sanitation and environmental modifications seemed to be a positive complement to the chemotherapy program. These modifications include the relatively high prevalence of biogas toilets and the recognition that bovines are reservoir hosts whose stool needs to be managed. Snail control using molluscicides, which was not specifically addressed in the survey, was another complementary strategy in the recent control work in this region. Control of the intermediate host likely had an effect on reducing infection, at least in the short term. On the whole, given the persistence of many risk factors, there is concern that infection levels may return to their previous levels. Hence, surveillance and treatment of infection will remain important and necessary strategies to maintaining low levels of infection in this area. In the short term, at least with the low levels of infection in this region, praziquantel remains an effective drug for treatment.

## Figures and Tables

**Table 1:** Infection prevalence by age and gender

Age (years)	Examined			Infected			Prevalence of infection		
	Female	Male	*N*	Female	Male	*N*	Female (%)	Male (%)	Percent
4–9	51	62	113	0	4	4	0.0	6.5	3.5
10–19	278	313	591	5	24	29	1.8	7.7	4.9
20–29	179	157	336	9	9	18	5.0	5.7	5.4
30–39	317	303	620	20	25	45	6.3	8.3	7.3
40–49	433	351	784	32	14	46	7.4	4.0	5.9
50–59	247	240	487	11	10	21	4.5	4.2	4.3
60+	93	129	222	4	15	19	4.3	11.6	8.6
Total	1,598	1,555	3,153	81	101	182	5.1	6.5	5.8

**Table 2:** Infection prevalence by village in 2009 and in a subset of villages in 2000 and 2002 surveys

Administrative village	Natural village	Examined	Hatch positive	Prevalence of infection (%)	Prevalence of infection in 2002 (%)	Prevalence of infection in 2000 (%)
1	1	163	10	6.1		
1	2	153	5	3.3		
1	3	110	3	2.7		
1	4	115	9	7.8		
1	5	91	11	12		
1	6	115	8	7.0	32	65
1	7	73	5	6.8		
1	8	53	5	9.4		
1	9	90	4	4.4		
1	10	71	12	17		
1	11	60	6	10		
1	12	94	6	6.4		
1	13	79	13	16		
Village 1 total		1,267	97	7.7		
2	1	43	2	4.7		
2	2	108	1	0.9		
2	3	89	1	1.1		
2	4	103	2	1.9		
2	5	85	4	4.7	28	68
2	6	118	8	6.8		
2	7	98	3	3.1		
2	8	159	0	0.0		
2	9	47	0	0.0		
2	10	50	2	4.0		
Village 2 total		900	23	2.6		
3	1	94	2	2.1		
3	2	140	4	2.9		
3	3	164	11	6.7	23	44
3	4	149	8	5.4		
3	5	103	7	6.8		
3	6	105	9	8.6		
3	7	123	8	6.5	38	73
3	9	56	3	5.4		
3	10	74	1	1.4		
3	11	94	12	13		
Village 3 total		1,102	65	5.9		
Total		3,269	185	5.7		

Difference between years was significant (Fisher's exact test; *P* < 0.001).

**Table 3:** Miracidial susceptibility to different praziquantel concentrations and exposure times

Praziquantel concentration (M)	Miracidia number	Shape unchanged after exposure period (in minutes)							
		1	2	3	4	5	7	9	10
Control (water)	5	100%	100%	100%	100%	100%	100%	100%	100%
DMSO 10^−3^	5	100%	100%	100%	100%	100%	100%	100%	100%
10^−8^	5	100%	100%	100%	100%	100%	100%	80%	60%
10^−7^	21	100%	95%	86%	81%	71%	57%	48%	33%
10^−6^	24	96%	71%	50%	33%	29%	21%	13%	13%
10^−5^	5	100%	100%	40%	20%	20%	20%	20%	20%

**Table 4:** Demographic characteristics of resistance factors survey participants in each of the three administrative villages in the township

	Administrative village 1	Administrative village 2	Administrative village 3
*N*	20	20	20
Gender (% female)	35	40	75
Mean age in years	42	46	39
SD	10	11	9
Range	21–63	13–61	23–56
Economic status (%)			
Low	10	35	25
Medium	85	65	75
High	5	0	0
Educational attainment (%)			
None	5	5	10
Less than elementary	5	20	20
Elementary	25	50	25
Middle	60	20	45
High	5	5	0
Occupation (multiple occupations allowed; %)			
Farmer	80	95	95
Student	0	0	0
Businessman	20	0	0
Local government official	0	0	0
Domestic worker	40	55	50

**Table 5:** Responses from the resistance risk factors survey

	Administrative village 1		Administrative village 2		Administrative village 3		All	*P*
	Ratio	Percent	Ratio	Percent	Ratio	Percent		
A. Roles and responsibilities								
1. Who is responsible for diagnosis and treatment?								0.058
Schistosomiasis control station	15/20	75%	20/20	100%	18/20	90%	88%	
Village doctor	5/20	25%	0/20	0%	2/20	10%	12%	
Village or township leader	0/20	0%	0/20	0%	0/20	0%	0%	
2. Who is responsible for reducing the risk of infection?								0.002
Schistosomiasis control station	14/20	70%	19/19	100%	19/20	95%	88%	
Village doctor	6/20	30%	0/19	0%	0/20	0%	10%	
Village or township leader	0/20	0%	0/19	0%	1/20	5%	2%	
3. If previously infected, who do you go to for treatment?								0.009
Schistosomiasis control station	0/6	0%	0/15	0%	0/6	0%	0%	
Village doctor	1/6	17%	10/15	67%	6/6	100%	63%	
Village or township leader	5/6	83%	5/15	33%	0/6	0%	37%	
4. If previously infected, do you need a reminder to take the drug?								0.002
No, I do not need a reminder	6/6	100%	3/15	20%	2/6	33%	41%	
Yes, village leader reminds me	0/6	0%	12/15	80%	4/6	67%	59%	
B. History of infection and treatment								
1. Has history of infection	7/18	39%	15/20	75%	6/19	32%	49%	0.015
2. How many times have you taken the drug in the past 3 years?								0.922
One time	0/6	0%	1/13	8%	0/6	0%	4%	
Two times	1/6	17%	1/13	8%	0/6	0%	8%	
Three times	4/6	67%	10/13	77%	6/6	100%	80%	
Four times	1/6	17%	1/13	8%	0/6	0%	8%	
C. Drug treatment experience								
1. Has side effects from treatment	3/6	50%	10/15	67%	1/6	17%	52%	0.133
2. Side effects affect work	0/3	0%	4/9	44%	0/1	0%	31%	0.648
3. It is convenient to get treatment	6/6	100%	15/15	100%	5/5	100%	100%	–
4. Consumes the drug immediately*	6/6	100%	8/15	53%	3/5	60%	65%	0.124
5. It is important to consume the entire dose	6/6	100%	14/15	93%	5/5	100%	96%	1
D. Knowledge and prevention of schistosomiasis								
1. How is schistosomiasis transmitted? (multiple answers allowed)								
Contact with irrigation ditches, lakes, and ponds	12/20	60%	19/20	95%	18/20	90%	82%	0.019
Swimming	6/20	30%	13/20	65%	10/20	50%	48%	0.101
Drinking unsanitary water	4/20	20%	10/20	50%	9/20	45%	38%	0.127
Something related to snails	5/20	25%	9/20	45%	9/20	45%	38%	0.36
Do not know	4/20	20%	0/20	0%	1/20	5%	8%	0.115
2. How can you prevent infection? (multiple answers allowed)								
Wear rain boots in infection risk areas	14/20	70%	13/20	65%	16/20	80%	72%	0.675
Wear gloves	6/20	30%	13/20	65%	9/20	45%	47%	0.1
Preventative medicine/plaster	0/20	0%	0/20	0%	1/20	5%	2%	1
No way	5/20	25%	6/20	30%	3/20	15%	23%	0.641
E. Clean water and sanitation								
1. What kind of toilet do you have?								0.033
Simple toilet	7/20	35%	6/19	32%	9/15	60%	41%	
Sanitary toilet	3/20	15%	0/19	0%	3/15	20%	11%	
Biogas toilet	10/20	50%	13/19	68%	3/15	20%	48%	
2. Shares a toilet with other households	5/18	28%	1/12	8%	0/13	0%	14%	0.068
3. Does not mind going to the bathroom outdoors in the wild	9/20	45%	17/20	85%	11/20	55%	62%	0.026
4. Aware that schistosomiasis is transmitted by cow stool	6/6	100%	12/12	100%	4/4	100%	100%	–
5. Believes cow stool should be removed	4/6	67%	10/12	83%	2/3	67%	76%	0.643
6. Has access to clean piped water	20/20	100%	20/20	100%	19/19	100%	100%	–
7. How do you use clean water? (multiple answers allowed)								
Cooking and drinking	20/20	100%	20/20	100%	19/19	100%	100%	–
Washing vegetables	20/20	100%	20/20	100%	16/19	84%	95%	0.03
Bathing	20/20	100%	17/20	85%	15/19	79%	88%	0.084
Washing laundry	16/20	80%	17/20	85%	15/19	79%	81%	0.919
8. Has contact with unsanitary water	17/20	85%	18/19	95%	17/20	85%	88%	0.68
9. Where do you contact unsanitary water? (multiple answers allowed)								
River	7/17	41%	14/18	78%	6/17	35%	52%	0.026
Pond	4/17	24%	12/18	67%	7/17	41%	44%	0.04
Ditches	14/17	82%	17/18	94%	17/17	100%	92%	0.205
Reservoirs	0/17	0%	1/18	6%	0/17	0%	2%	1
10. How do you use unsanitary water? (multiple answers allowed)								
Cooking and drinking	4/17	24%	12/18	67%	6/17	35%	42%	0.032
Washing vegetables	4/17	24%	12/18	67%	8/17	47%	46%	0.04
Bathing	4/17	24%	13/18	72%	7/17	41%	46%	0.015
Washing laundry	7/17	41%	14/18	78%	9/17	53%	58%	0.088

* Those that responded no and said that they consumed the drug at night.

**Table 6:** Risk factors for self-reported history of infection

	Age-, sex-, and income-adjusted			Unadjusted		
	OR	*P*	95% CI	OR	*P*	95% CI
Village doctor is responsible for treatment	0.27	< 0.001	0.18–0.42	0.23	< 0.001	0.15–0.36
Village doctor is responsible for reducing the risk of infection	0.23	< 0.001	0.15–0.33	0.19	< 0.001	0.14–0.28
Number of treatments in the past 3 years	0.48	0.254	0.13–1.70	0.75	0.043	0.56–0.99
Reported treatment side effects	2.52	0.426	0.26–24.4	2.59	0.396	0.29–23.3
Consumes drug immediately	2.12	0.398	0.37–12.2	1.38	0.412	0.64–3.01
Poor knowledge (drinking is responsible for infection)	0.74	0.512	0.30–1.81	0.80	0.19	0.58–1.12
Poor knowledge (does not know how infection occurs)	1.21	0.82	0.23–6.42	0.95	0.935	0.30–3.03
Boots prevent infection	1.06	0.937	0.24–4.60	0.90	0.872	0.26–3.18
Gloves prevent infection	3.63	0.027	1.16–11.4	2.90	0.177	0.62–13.6
No way to prevent infection	1.15	0.88	0.19–6.96	1.34	0.638	0.39–4.62
Sanitary toilet	0.35	0.12	0.09–1.31	0.33	0.009	0.14–0.76
Biogas toilet	0.88	0.891	0.14–5.54	0.89	0.858	0.24–3.23
Does not mind going to bathroom outdoors in the wild	1.34	0.76	0.21–8.67	1.30	0.706	0.33–5.18
Contacts unsanitary water	1.64	0.656	0.19–14.4	1.73	0.553	0.28–10.6
Contacts unsanitary water (river)	2.08	0.376	0.41–10.4	1.85	0.363	0.49–6.94
Contacts unsanitary water (pond)	4.58	0.005	1.58–13.3	2.96	0.001	1.54–5.71
Contacts unsanitary water (ditch)	0.95	0.92	0.37–2.46	1.11	0.837	0.43–2.87
Contacts unsanitary water (cooking and drinking)	3.46	0.004	1.48–8.07	2.44	< 0.001	1.86–3.22
Contacts unsanitary water (washing vegetables)	3.61	0.012	1.32–9.83	2.47	< 0.001	1.61–3.77
Contacts unsanitary water (bathing)	5.21	0.001	1.92–14.1	3.55	< 0.001	2.17–5.79
Contacts unsanitary water (washing laundry)	1.90	0.442	0.37–9.75	1.67	0.399	0.51–5.50
